# Analysis of tumor-infiltrating exhausted T cells highlights IL-6 and PD1 blockade as a combined immunotherapy strategy for non-small cell lung cancer

**DOI:** 10.3389/fimmu.2025.1486329

**Published:** 2025-02-11

**Authors:** Lulu Zhang, Xiyuan Guo, Xiaoke Sun, Jue Liao, Qin Liu, Yingchun Ye, Zhihui Yang, Ratchada Cressey, Qing He, Qing Yuan

**Affiliations:** ^1^ Public Center of Experimental Technology, The School of Basic Medical Sciences, Southwest Medical University, Luzhou, Sichuan, China; ^2^ Key Laboratory of Medical Electrophysiology of the Ministry of Education, Medical Electrophysiological Key Laboratory of Sichuan Province, Institute of Cardiovascular Research, Southwest Medical University, Luzhou, China; ^3^ Department of Head and Neck Oncology, West China Hospital of Sichuan University, Chengdu, Sichuan, China; ^4^ Division of Clinical Chemistry, Department of Medical Technology, Faculty of Department of Medical Technology, Faculty of Associated Medical Sciences, Chiang Mai University, Chiang Mai, Thailand; ^5^ Institute of Nuclear Medicine, Nuclear Medicine and Molecular Imaging Key Laboratory of Sichuan Province, Southwest Medical University, Luzhou, Sichuan, China; ^6^ Department of Pathology, The Affiliated Hospital of Southwest Medical University, Luzhou, Sichuan, China; ^7^ Blood Distribution Department Nanjing Red Cross Blood Center, Nanjing, Jiangsu, China

**Keywords:** exhausted CD8+T cells, non-small cell lung cancer, interleukin-6, PD1, combined blockade

## Abstract

**Objective:**

Given the limitations of immunotherapy for treating non-small cell lung cancer (NSCLC), we investigated the phenotype and function of exhausted CD8^+^T cells and analyzed a novel combination immunotherapy to restore the effector killing function of tumor-infiltrating CD8^+^T lymphocyte (TIL).

**Methods:**

We examined the expression and function of immunosuppressive molecules on CD8^+^T cells of peripheral blood mononuclear cells (PBMCs) and TILs by using prospectively collected peripheral blood, pleural effusions, and tumor tissues from patients with NSCLC and correlated the results with clinical data. We then evaluated the effect of interleukin 6 (IL-6) stimulation on CD8^+^T cells. Finally, we assessed the effects of combined blockade of PD1 and IL-6 on macrophage recruitment in a zebrafish macrophage model and CD8^+^ T cell function and tumor growth in PBMC humanized mouse model.

**Results:**

The expression of exhaustion markers on CD8^+^ T cells was found to be notably higher in both tumor and paraneoplastic tissues compared to peripheral blood. Furthermore, the degree of CD8^+^ T cell exhaustion exhibited a progressive increase with proximity to the tumor. When CD8^+^ T cells from peripheral blood and tumor tissues of NSCLC patients were stimulated with IL-6, the expression level of exhaustion markers, especially PD1, was further elevated. In the *in vitro* experiment, the combined inhibition of IL-6 and PD1 substantially enhanced the effector killing function of CD8^+^ T cells in NSCLC pleural effusion samples. In a macrophage-labeled zebrafish model, combined blockade of IL-6 and PD1 enhanced the recruitment of macrophages. In PBMC humanized mouse model, combined blockade of IL-6 and PD1 enhanced the inhibition of tumor growth.

**Conclusion:**

Our data suggest that CD8^+^ T cells in NSCLC patients were in a state of exhaustion and combined blockade of IL-6 and PD1 to restore CD8^+^ T cell function to inhibit tumor growth may be an effective clinical strategy for the treatment of NSCLC.

## 1 Introduction

Lung cancer is one of the most common malignant tumors and the leading cause of cancer-related deaths worldwide (1). This disease has two main histological types: small cell lung cancer (SCLC) and non-small cell lung cancer (NSCLC). Of these histological types, NSCLC accounts for approximately 80% of all lung cancers, and it is the main primary pathological type of lung cancer (2). Usually, because of the late onset of symptoms, most patients are diagnosed at advanced stages of lung cancer, and the lack of effective interventions and specific molecular targets is the primary reason for the treatment failure of patients with NSCLC, these patients have a 5-year survival rate of approximately 10-20%. Hence, it is important to find novel molecular therapeutic targets for NSCLC (3).

Cytotoxic CD8^+^T cells play a key role in antitumor immunity, however, because of tumor microenvironment (TME) suppression and prolonged exposure to antigens, tumor-specific effector CD8^+^ T cells readily differentiate to a stage known as “T cell exhaustion” (4, 5). CD8^+^ T cells in the exhaustion phase are distinct from functional effector T cells and memory T cells. The two main characteristics of CD8^+^ T cells in the exhaustion phase are the progressive loss of effector cytokine production and the sustained expression of various inhibitory receptors (IRs), such as PD1, LAG-3, and Tim-3 (6, 7). The intensity and number of immunosuppressive receptors expressed by exhausted T cells are positively correlated with exhaustion severity, and some newly discovered immune molecules, such as CD352 and CD39, are also expressed in varying degrees in exhausted CD8^+^T cells (8, 9). Additionally, exhausted CD8^+^T cells have a unique transcriptional program (low TCF1 expression and a high Eomes and TOX expression) and proliferative state (10–12). Most importantly, the exhaustion of CD8^+^T cells leads to poor cancer immunotherapy.

PD1 expression determines the dysfunction status of CD8^+^T cells. It negatively regulates the signaling of T cell receptor/CD28 and decreases T cell proliferation and cytokine secretion following binding to ligands (PD-L1 and PD-L2). The increased expression of PD1 in circulating T cells associated the with poor prognosis of patients (13, 14). The development of cancer immunotherapy has drastically changed the landscape of tumor therapy. The use of PD1 antibodies has reduced tumor progression and provided long-term clinical benefits to tumor patients (15). However, only a small percentage of patients benefit from this therapeutic approach, and relapse frequently occurs due to various resistance mechanisms. The limitation of immune checkpoint inhibitor (ICI) monotherapy in most cancer patients lies in the complex TME, where multiple immunosuppressive factors are present at each step of the cancer immune cycle (16, 17). For effective tumor control and eradication, combination therapy and/or targeting of additional immune checkpoints appears to be a critical approach.

Over the past few decades, cytokines and cytokine receptors have been extensively studied as cancer targets or cancer therapies (18). CD8^+^T cells in TME may fail to respond, in part because certain cytokines are involved in promoting exhaustion (19–21). IL-6 is an inflammatory cytokine involved in various biological processes, including immune dysregulation and cancer (22). IL-6 promotes tumor cell survival, and the blockade of IL-6 reduces the side effects of ICIs while enhancing the antitumor immune response, leading to dual effects (23, 24). In patients undergoing treatment with ICIs for metastatic melanoma are linked to an unfavorable prognosis among those with tumors. Those with elevated serum IL-6 levels display an increased likelihood of developing brain metastases, making IL-6 a valuable predictive marker for brain metastasis in NSCLC patients (25). Nonetheless, limited research has explored the impact of IL-6 on the characteristics and function of exhausted T cells in NSCLC. Specifically, the consequences of combined blockade with PD1 on the function of exhausted T cells have not been fully clarified.

To address these issues, we investigated the phenotypic and functional changes of CD8^+^ T cells in NSCLC patients. We propose to restore the function of exhausted CD8^+^ T cells in the tumor microenvironment by combined blockade of PD1 and IL-6 thereby inhibiting tumor growth. Our study will contribute to a deeper understanding of T cell exhaustion and provide potential therapeutic strategies for NSCLC.

## 2 Methods

### 2.1 Preparation of mononuclear cells from human peripheral blood and pleural fluid

The collection of peripheral blood and pleural effusions from patients with NSCLC was approved by the Clinical Trial Ethics Committee of the Affiliated Hospital of Southwest Medical University (Approval No. KY2019276). Human peripheral blood mononuclear cells (PBMCs) and pleural effusion mononuclear cells (PEMCs) were isolated from peripheral blood and pleural effusions by using density gradient centrifugation (Cytiva, Sweden), respectively.

### 2.2 Preparation of single-cell suspension of human tumor tissues

Peripheral blood, paraneoplastic and cancerous tissues were collected from patients with primary, early stage non-small cell lung cancer (pathologically confirmed after surgery), which was approved by the Clinical Trial Ethics Committee of the Affiliated Hospital of Southwest Medical University (Approval No. KY2019276). Fresh tumors and paracancerous tissues from NSCLC patients were collected in sterile RPMI-1640 medium (Gibco, USA). Paired fresh tumor and paracancerous tissue samples were cut into small pieces to prepare single-cell suspensions for analyzing tissue-infiltrating lymphocytes (TILs). The tissues were washed with 4°C PBS, ground with a tissue grinder (Miltenyi, Germany), and gently processed with a MACS Dissociator (Miltenyi, Germany), to obtain single-cell suspensions. Erythrocytes were lysed after diluting the single-cell suspension with PBS and filtering through a 70-µm filter. The prepared cells were used for *in vitro* assays or analyzed by flow cytometry.

### 2.3 Flow cytometry

A total of 1 × 10^6^ cells were suspended in 100 µL of PBS and exposed to an appropriate quantity of fluorescent labeling antibodies. This mixture was then incubated for 30 minutes at room temperature. In order to detect intracellular cytokines, the PBMCs that had been isolated were resuspended in RPMI 1640 medium containing 10% fetal bovine serum, penicillin (100 units/mL), and streptomycin (100 µg/mL). The cells, at a concentration of 10^6^ cells/mL, were cultured in 12-well plates and stimulated with PMA/ionomycin (including Brefeldin A) at a concentration of 2 µL/mL (BioLegend, USA). Subsequently, the cells were incubated at 37°C in an atmosphere of 5% CO_2_ for a duration of 6 h. To perform intracellular staining, the cells were fixed and permeabilized as per the manufacturer’s instructions using the Foxp3 staining buffer set (eBioscience, USA) for a period of 20 min.

The Apoptosis Membrane Linker Protein V Apoptosis Detection Kit (BD Biosciences, USA) based on FITC/PE was used for T cells.

Cells were analyzed by a flow cytometer (BD Biosciences, USA), and the data were analyzed by FlowJo software. The antibodies used for flow cytometry are listed in **Supplementary Table 1**.

### 2.4 Cytokine assay

The levels of cytokines IL-6, IL-7, IL-10, and IL-17A in both plasma and tissue supernatants were assessed using flow cytometry (Cytometric Bead Array, CBA). For the magnetic bead assay, magnetic beads, standards, reagents, and samples were prepared following the manufacturer’s instructions (BD Biosciences, USA). To summarize, capture beads were introduced into the assay tube (50 µL/tube), combined with 50 µL of sample or standard, and incubated in the dark for 1 h. An additional 50 µL of corresponding PE-labeled antibodies were added, and the mixture was further incubated for 2 h at room temperature in the dark. Subsequently, the samples underwent washing with a wash buffer and were subjected to flow cytometry analysis. Data analysis was conducted using FCAP Array V3 software (BD Biosciences).

### 2.5 RNA isolation and quantitative real-time PCR assay

Tissues were ground and resuspended in 1 mL of RNAiso Plus solution (Takara, Japan). RNA extraction was performed using the Total RNA Extraction Kit (Takara, Japan) according to the manufacturer’s instructions. The extracted RNA was reverse-transcribed into cDNA using the Reverse Transcription Kit (Takara, Japan), with the cDNA serving as a template for amplification. All samples were tested in triplicate. Primers were designed using synthetic primers, and the sequences of upstream and downstream primers are detailed in **Supplementary Table 2**. GAPDH was employed as the internal reference gene, and the relative expression of cytokine genes was calculated using the 2-ΔΔCt method.

### 2.6 *Ex vivo* stimulation of IL-6

Human PBMCs and TILs were cultured in RPMI 1640 medium at the density of 10^6^ cells/mL and incubated with 20 ng/mL of IL-6 (PeproTech, USA) for 24 h at 37°C in 5% CO_2_.

### 2.7 IL-6 and PD1 blockade

In brief, 48-well round bottom plates were coated with 1 µg/mL anti-CD3 antibodies (BioLegend, USA) and left overnight at 4°C. The plates were washed thrice with PBS. PBMCs or TILs from NSCLC patients were placed in 400 µL of RPMI 1640 medium containing 1 µg/mL anti-CD28 antibodies (BioLegend, USA) supplemented with 10 µg/mL of nivolumab (Selleck, China) or IgG, and 10 µg/mL of anti-IL-6 antibodies (GLPBIO, USA) and incubated at 37° C in 5% CO_2_ for 72 h.

### 2.8 Isolation of CD8^+^ T cells and co-culture with tumor cells

A549 cells were cultured in RPMI 1640 medium supplemented with 10% fetal bovine serum and 1% penicillin-streptomycin mixture. CD8^+^ T cells were isolated and purified from healthy human peripheral blood PBMCs using the MojoSort™ Human CD8^+^ T cell Isolation Kit (BioLegend, USA). These CD8^+^ T cells were then cultured in RPMI 1640 medium with anti-CD3 (1 µg/mL) and anti-CD28 antibodies (1 µg/mL) (BioLegend, USA) and supplemented with nivolumab (10 µg/mL) (Selleck, China) or anti-IL-6 (10 µg/mL) antibodies. Cells were cultured at 37°C in 5% CO_2_ for 72 h and subsequently co-cultured with A549 cells at an effector-target ratio of 10:1 for 48 h to assess the apoptosis of A549 cells.

### 2.9 Eligibility criteria for participants

patients with untreated non-small cell lung cancer diagnosed clinically and approved by the Ethics Committee of the hospital, the subjects were informed and volunteered to participate in this study. The following patients are excluded: patients with acquired immunodeficiency syndrome; patients with autoimmune diseases such as type I diabetes mellitus, systemic lupus erythematosus, rheumatoid arthritis, etc.; and patients with severe damage to vital organs.

### 2.10 TISCH database analysis of exhausted T cells

The TISCH database (http://tisch.comp-genomics.org/) provides detailed cell type annotations at the single cell level, which helps to explore the TME in different cancer types. In this study, the TISCH database was applied to analyze the expression of exhausted T cells in NSCLC patients.

### 2.11 TCGA database analysis of IL-6

Overall survival (OS) was estimated using the Kaplan Meier method. All quantitative variables were expressed as mean ± standard deviation or median. Mann-Whitney U test was used to compare the median differences between the groups. Cut-off values for OS were determined using the receiver operating characteristic (ROC) curve. RNAseq data (level 3) and corresponding clinical information for NSCLC were obtained from TCGA (https://portal.gdc.com) database. IL-6 was analyzed for correlation with pathway scores by Spearman correlation. Kaplan-Meier curves, p-values and hazard ratios (HR) with 95% confidence intervals (CI) were obtained by logrank test and univariate Cox regression. All analytical methods and R packages were performed using R software version v4.0.3. p < 0.05 was considered statistically significant.

### 2.12 Western blot analysis

Protein expression of Tox (CST,USA) and Tcf-1 (CST,USA) were analyzed by Western blot. Cells were lysed in RIPA buffer (Santa Cruz Biotechnology, USA) with protease inhibitors (1:100) for 30 minutes on ice. Protein concentrations were determined using the Enhanced BCA Protein Assay Kit (Beyotime Biotechnology, China). Equal protein amounts (25 µg) were separated on 10% SDS-PAGE gels and transferred to NC membranes. Membranes were blocked with 5% BSA, incubated overnight with primary antibodies at 4°C, followed by HRP-conjugated secondary antibodies. β-actin was employed as the internal reference gene. Bands were visualized with chemiluminescence and quantified using ImageJ. The antibodies used for WB are listed in **Supplementary Table 3**.

### 2.13 Feeding and maintenance of zebrafish

Adult zebrafish (males and females) were maintained at 28°C with a light/dark cycle of 14/10 hpf according to the actual rearing license at zebrafish platform of public technology center of southwest medical university. Embryos were obtained by natural spawning and kept under 28°C incubator until 24 hpf. PTU (Macklin, China) was applied to improve transparency and E3 medium was changed daily.

Zebrafish used for spawning were 6-12 month old adult Tg (mpeg1:DsRed) CZ5 strain zebrafish with expressed macrophages labeled red (26). A549 was stained with Dio (Beyotime, USA). After embryos were cultured to 48 hpf and membranes were manually stripped, stained A549 cell suspensions and unstained healthy volunteer human PBMCs were co-injected into the zebrafish yolk sac space using a microinjector at a ratio of 9:1 (~400 cells/fish). Zebrafish with sufficient fluorescence of tumor cells were selected to be randomized into groups of at least 5 fish each. PBS was used as a negative control group, and anti-PD1, anti-IL-6, and a combination of anti-PD1 and anti-IL-6 were used as treatment groups. 1 nl of antibody or control was injected intravenously every day, and macrophage recruitment in image larvae were captured using a confocal microscopy (Olympus) two days after treatment. All experiments were approved by the Animal Research Ethics Committee of Southwest Medical University.

Data were analyzed using ImageJ software, Prism9 graphic software and SPSS 17.0. Statistical analyses were expressed as mean ± standard deviation. p< value of 0.05 was considered as statistically significant difference.

### 2.14 Mouse experiments

6-8-week-old female severely immunodeficient mice (NTG) were obtained from Chongqing Tengxin Huafu Laboratory Animal Sales Co. and placed in an SPF (Specific Pathogen Free) environment for 1 week for acclimatization. In this animal experiment, we adhered to animal ethics, and the treatment of mice strictly followed the criteria for the care and use of research animals and was authorized by Southwest Medical University’s Animal Ethics Committee. Each mouse was injected subcutaneously with A549 cells (3×10^6^). When the tumor volume reached 80mm^3^, each mouse was injected with 1×10^7^ healthy volunteer PBMCs in the tail vein. Three days after the injection, the mice were randomly divided into four groups of six mice each, with nivolumab, anti-IL-6, and nivolumab combined with anti-IL-6 antibody as the treatment groups, and IgG isotype control as the negative control group. The antibodies were injected from the tail vein of the mice every three days for 2 weeks, and the body weights and tumor sizes of the mice were recorded during the treatment. The formula used to compute tumor volume was tumor volume (mm3) = 1/2×length×width^2^. The mice were euthanized at the end of the treatment, and peripheral blood and tumors were taken for analysis.

### 2.15 Statistical methods

Statistical analysis was performed using SPSS version 26.0 software and GraphPad Prism version 8. Paired t-test and ANOVA were used for between-group comparisons for normally distributed and equal-variance data. Mann-Whitney U test was used to analyze differences between two independent samples for non-normally distributed variables, and the Kruskal-Wallis test was used to analyze differences between multiple independent samples. Spearman’s correlation coefficients were used to analyze the relationship between cellular levels and clinical or laboratory parameters. Differences were considered statistically significant at P < 0.05 (*P < 0.05, **P < 0.01, ***P < 0.001).

## 3 Results

### 3.1 Tumor-infiltrating CD8^+^T cells appear exhausted in patients with NSCLC

According to previous reports, the frequency and phenotype of circulating lymphocytes are important for the treatment outcomes of cancer patients and for those receiving ICI blockade therapy. In the present study, we compared the frequency of lymphocytes in peripheral blood PBMCs from healthy volunteers, peripheral blood PBMCs from NSCLC patients, and peri-tumor tissues and tumor tissues by flow cytometry. The peri-tumor tissues and tumor tissues were prepared into single-cell suspensions by using a single-cell processor.

**Figure 1:**
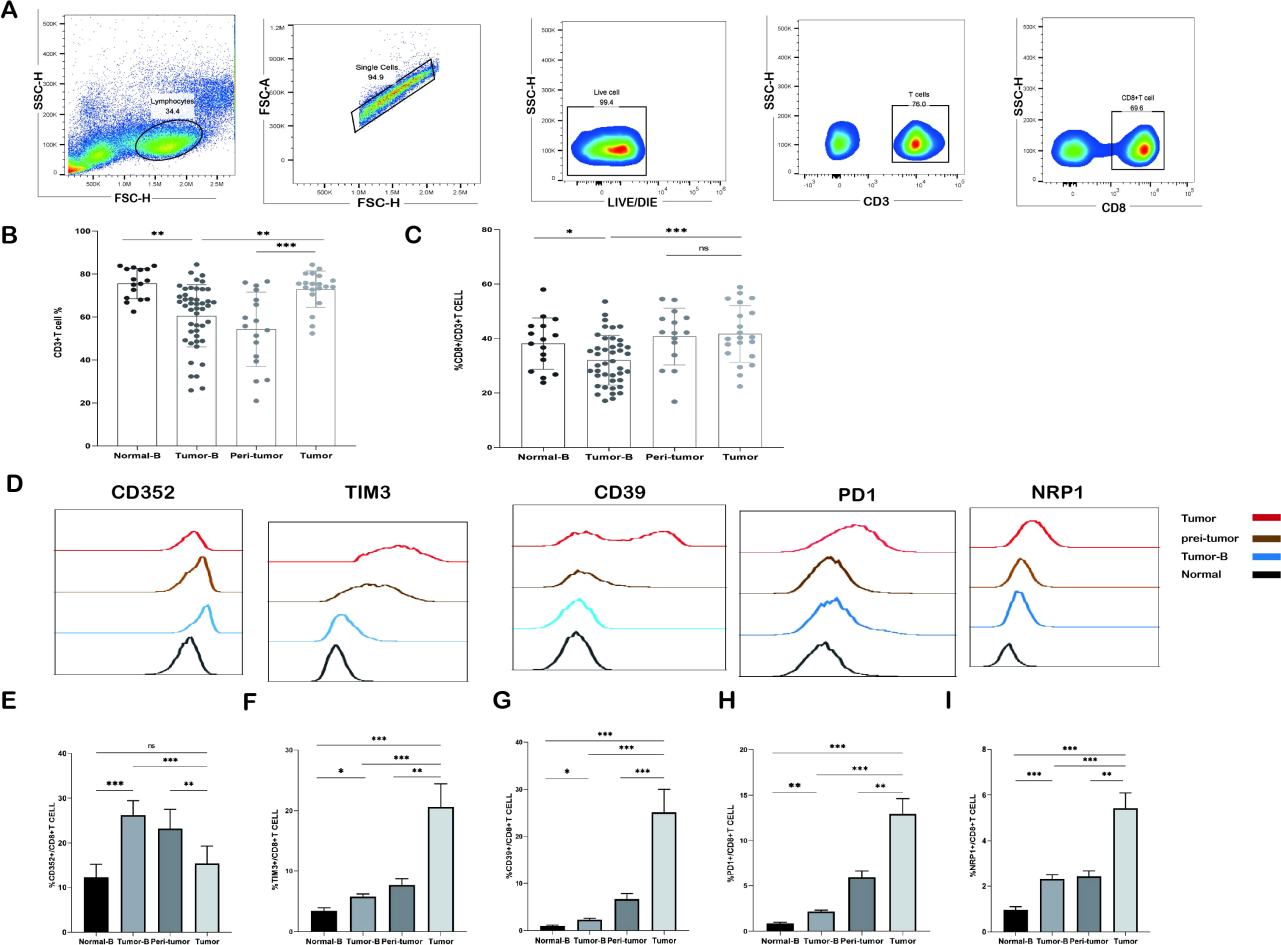
Frequency of CD3^+^ T cells and CD8^+^ T cells detected by flow cytometry. **(A)** Gating strategy for identifying CD3^+^ T cells and their subpopulations by flow cytometry. **(B)** Comparison of the frequency distribution and pairwise distribution of CD3^+^ T cells and CD8^+^ T cells in the peripheral blood of the Normal-B group (n = 16), Tumor-B group (n = 46), peri-tumor group (n = 17), and Tumor group (n = 20). **(C)** Frequency distribution and pairwise comparison of CD8^+^ T cells in peripheral blood in the Normal-B group (n = 16), Tumor-B group (n = 46), peri-tumor group (n = 17), and Tumor group (n = 20). **(D)** Typical staining of CD352, TIM3, CD39, PD1, and NRP1 expression in CD8^+^ T cells in the Normal-B, Tumor-B, and Tumor groups. **(E-I)** Frequency plots of CD352, TIM3, CD39, PD1, and NRP1 expression in CD8^+^ T cells in the Normal-B, Tumor-B, peri-tumor, and Tumor groups. Normal-B, Tumor-B, peri-tumor, and Tumor groups are peripheral blood PBMC group of healthy volunteers, peripheral blood PBMC group of patients with non-small cell lung cancer (NSCLC), paracancerous tissue group of patients with NSCLC, and cancerous tissue group of patients with NSCLC, respectively. ns, not statistically significant. *P < 0.05, **P < 0.01, ***P < 0.001.

The analysis results are shown in **Figure 1A**. The frequency of CD3^+^ T cells in peripheral blood PBMCs (Tumor B) was decreased in NSCLC patients as compared to that in the peripheral blood of the control group comprising healthy volunteers (Normal-B) (P < 0.01). CD3^+^ T cells were significantly increased in tumor tissues as compared to that in peri-tumor tissues (P < 0.001). Interestingly, CD3^+^T cell level in peripheral blood PBMCs was also significantly reduced in tumor patients as compared to that in tumor tissues (P < 0.01) (**Figure 1B**). Therefore, we compared changes in the proportion of CD8^+^ T cells in peripheral blood PBMCs and tumor tissues and found that CD8^+^ T cell proportion was also the highest in tumor tissues (P < 0.001) (**Figure 1C**).

In the following experiment, we explain why the increase in the number of infiltrating CD3^+^ T cells and CD8^+^ T cells in tumor tissues did not exert proper immunologic cytotoxicity. A possibility is the exhaustion of the function of CD8^+^ T cells. First, we analyzed the expression of the immunosuppressive molecules CD352, TIM3, CD39, PD1, and NRP1. The expression levels of these molecules were significantly upregulated in CD8^+^ T cells in peripheral blood, peri-tumor tissues, and tumor tissues of tumor patients as compared to that in peripheral blood PBMCs of healthy controls (**Figures 1D-I**); this finding suggests that CD8^+^ T cells may have been exhausted in NSCLC patients. We compared the frequency of CD8^+^ T cells expressing exhausted cell markers in the peripheral blood PBMCs and tissues of different NSCLC patients. TIM3, CD39, PD1, and NRP1 in CD8^+^ T cells were significantly elevated in peri-tumor tissues as compared to those in the peripheral blood PBMCs of NSCLC patients and further elevated in tumor tissues (P < 0.001). Interestingly, the frequency of CD352^+^CD8^+^ T cells showed a distinct trend: the expression frequency of CD352^+^CD8^+^ T cells in tumor tissues was significantly lower than that in the paraneoplastic tissues (P < 0.01), whereas the frequency of CD352^+^CD8^+^ T cells in the peripheral blood was the highest as compared to that in tumor tissues and peri-tumor tissues (P < 0.001).

### 3.2 Progressive exhaustion of CD8^+^T cells in the TME

**Figure 2:**
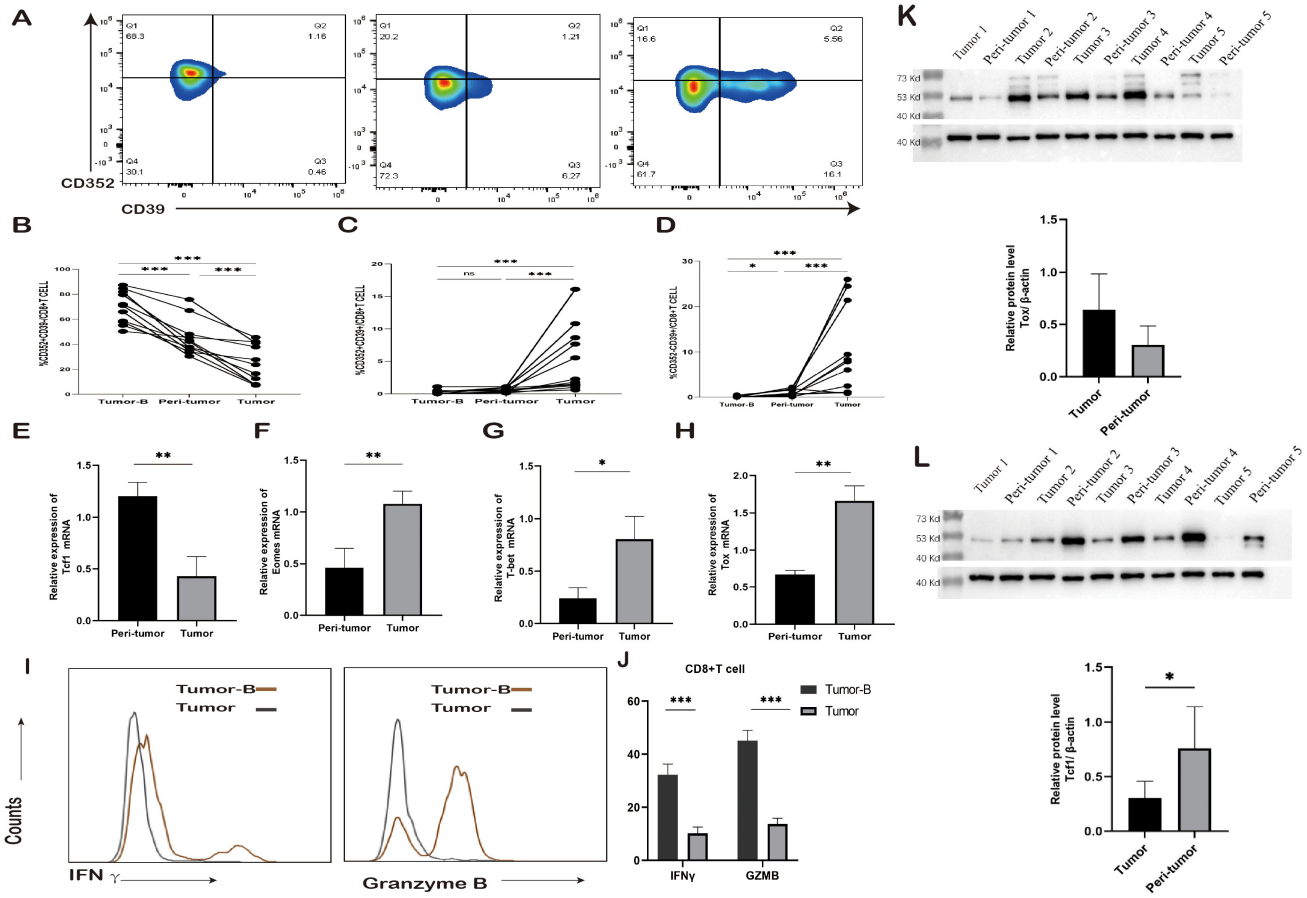
Changes in exhausted CD8^+^ T cell subsets. **(A)** Typical staining and expression frequency of CD352 and CD39 on CD8^+^ T cells in the Tumor-B, peri-tumor, and Tumor groups of the same lung cancer patients. Horizontal lines represent mean values. **(B-D)** Comparison of the expression frequency of CD352 and CD39 in CD8^+^ T cells in the Tumor-B, peri-tumor, and Tumor groups of the same lung cancer patients. **(E)** The mRNA expression level of Tcf1 in the peri-tumor and Tumor groups. **(F)** The mRNA expression level of Emoes in the peri-tumor and Tumor groups. **(G)** The mRNA expression level of T-bet in the peri-tumor and Tumor groups. **(H)** The mRNA expression level of Tox in the peri-tumor and Tumor groups. **(I)** Typical staining of CD8^+^ T cells producing IFN-γ and GZMB in the Tumor-B and Tumor groups. **(J)** Comparison of the frequency of IFN-γ and GZMB production by CD8^+^ T cells in the Tumor-B and Tumor groups. **(K)**The protein expression level of Tcf1 in the peri-tumor and Tumor groups. **(L)** The protein expression level of Tox in the peri-tumor and Tumor groups. The Tumor-B group was the peripheral blood PBMC group of patients with non-small cell lung cancer. The peri-tumor and Tumor groups were the paracancerous tissue group and cancer tissue group of patients with non-small cell lung cancer, respectively. ns, not statistically significant. *P < 0.05, **P < 0.01, ***P < 0.001.

T cells gradually move toward the exhaustion state after stimulation by tumor antigens. This process does not occur overnight. Previous studies have shown that different subpopulation states of exhausted T cells may exist. To gain a deeper insight into the exhaustion status of CD8^+^ T cells, an analysis was conducted on the expression frequency of CD352 and CD39 on CD8^+^ T cells in both PBMCs and tissues obtained from the same tumor patient (**Figure 2A**). The frequency of CD352^+^CD39^+^ T cells and CD352^-^CD8^+^ T cells was notably higher in tumor tissues, followed by peri-tumor tissues, while the lowest expression frequency was observed in the peripheral blood PBMCs of tumor patients. Notably, the peripheral blood PBMCs exhibited the highest percentage of CD352^+^CD39^+^CD8^+^ T cells (**Figures 2B-D**). This observation aligns with previous research findings, suggesting that CD352 may function as an initial marker of exhaustion for CD8^+^ T cells. Furthermore, an assessment of mRNA expression levels was carried out in both peri-tumor tissues and cancerous tissues within the same study group. In comparison to peri-tumor tissues, tumor tissues exhibited a significant reduction in the mRNA level of the transcription factor Tcf1 (**Figure 2E**). Conversely, the mRNA expression levels of Emoes, T-bet, and TOX, three transcription factors associated with terminal exhaustion, were elevated (**Figures 2G-H**). Protein analysis showed that the level of TCF1 in peri-tumor was significantly higher than that in tumor tissue, and TOX expression level in tumor tissue was higher than that in peri-tumor, which was consistent with transcriptome expression level (**Figures 2K-L**). This finding indicates that CD8^+^ T cells in the TME effectively transitioned from initial exhaustion to terminal exhaustion, and the degree of T cell exhaustion gradually deepened with proximity to the TME.

Subsequently, an investigation was conducted regarding the cytokine-producing capabilities of CD8^+^ T cells. Peripheral blood PBMCs and tumor tissue cells were isolated from 10 NSCLC patients and subjected to stimulation with phorbol 12-myristate 13-acetate (PMA) and ionomycin in the presence of Brefeldin A. The flow cytometry assay was employed to measure the levels of cytokine production by CD8^+^ T cells. The findings revealed that in comparison to peripheral blood PBMCs, the secretion of IFN-γ and GZMB by CD8^+^ T cells was notably diminished in tumor tissues (**Figures 2I, J**).

Granzyme B can enter target cells and activate the caspase cascade reaction, promptly inducing DNA breaks within the target cells and leading to their swift apoptosis. Furthermore, IFN-γ regulates the expression and secretion of CXCL9, CXCL10, and CXCL11, along with their cognate receptor CXCR3 in various immune cells such as T cells, NK cells, monocytes, DCs, and cancer cells. This regulation occurs through transcriptional control and promotes the migration of immune cells toward the TME. Consequently, it enhances the chemotaxis of activated CTLs towards the TME, resulting in heightened cytotoxicity and restriction of tumor growth. In summary, IFN-γ and GZMB play a pivotal role in antitumor therapy. These findings suggest that as CD8^+^ T cell exhaustion intensifies, the killing capacity of effector cells diminishes.

### 3.3 Increased inflammatory factors in the TME

**Figure 3:**
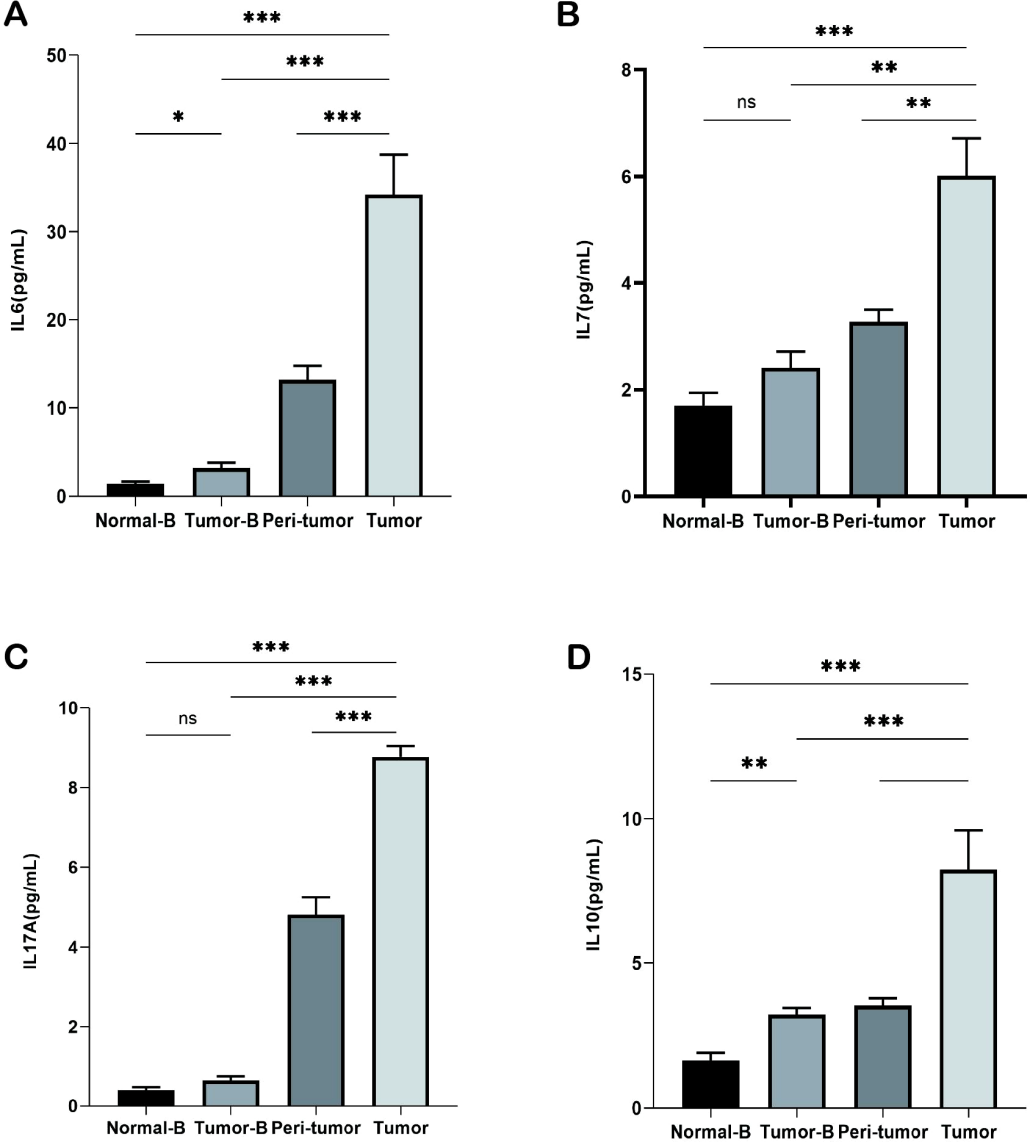
Detection of cytokines in plasma and tissue homogenization supernatants. **(A)** Detection of IL-6 levels in the Normal-B, Tumor-B, peri-tumor, and Tumor groups. **(B)** Detection of IL-7 levels in the Normal-B, Tumor-B, peri-tumor, and Tumor groups. **(C)** Detection of IL-17A levels in the Normal-B, Tumor-B, peri-tumor, and Tumor groups. **(D)** Detection of IL-10 levels in the Normal-B, Tumor-B, peri-tumor, and Tumor groups. Note: The Normal-B, Tumor-B, peri-tumor, and Tumor groups were the peripheral blood PBMC group of healthy volunteers, the peripheral blood PBMC group of non-small cell lung cancer, and the peritumor tissue and the cancerous tissue groups of patients with non-small cell lung cancer, respectively. ns, not statistically significant. *P < 0.05, **P < 0.01, ***P < 0.001.

Inflammatory factors affect immune surveillance and response to cancer treatment in the early stages of cancer. At least 20% of all cancers are associated with persistent infection and chronic inflammation. Cancers that do not occur because of chronic inflammation also exhibit extensive inflammatory infiltration. Therefore, we assessed changes in cytokine levels in plasma and in the homogenization supernatants of cancer and paracancerous tissues. IL-6, IL-7, IL-10, and IL-17A levels were elevated in the plasma of lung cancer patients as compared to those in healthy controls; thus, indicating higher concentrations of inflammatory mediators in patients. A comparison of changes in cytokine levels in plasma and tissue homogenization supernatants from different lung cancer patients revealed that the IL-6, IL-7, IL-10, and IL-17A expression levels were particularly elevated in tumor tissues and were the lowest in plasma supernatants (**Figures 3A-D**); this finding indicated that the closer the TME, the more pronounced is the inflammatory response.

### 3.4 Correlation between high expression of immunosuppressive molecules and CD8^+^T cell exhaustion

**Figure 4:**
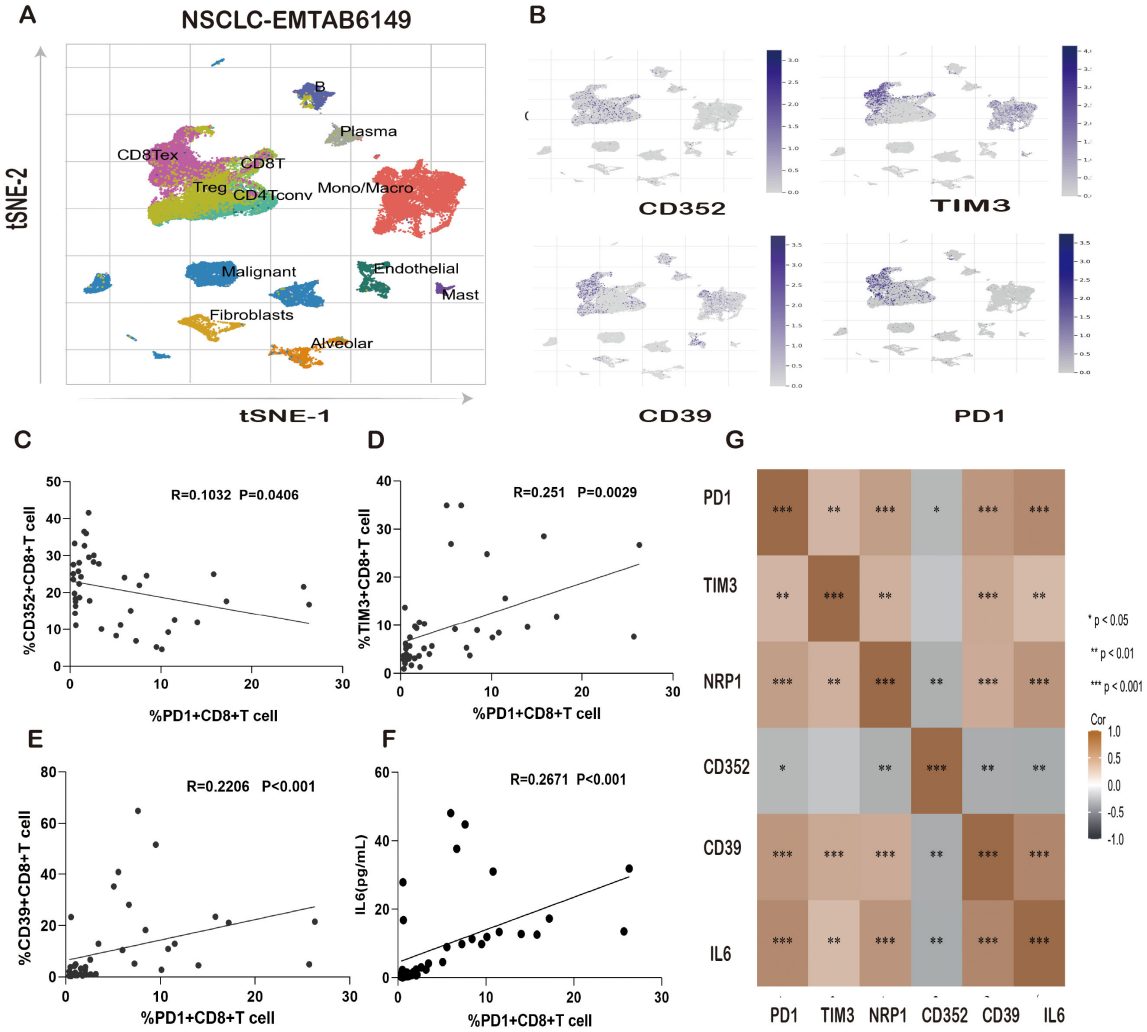
Correlation of IL-6 and PD-1 expression levels with T-cell exhaustion markers in lung cancer patients. **(A)** Distribution of immune cells in the NSCLC single-cell sequencing data (EMTAB6149). **(B)** Distribution of CD352, TIM3, CD39, and PD1 expression in the NSCLC single-cell sequencing data (EMTAB6149). **(C)** Correlation of the PD1^+^CD8^+^T-cell ratio with the CD352^+^CD8^+^ T-cell ratio in lung cancer patients. **(D)** Correlation between the PD1^+^ CD8^+^ T-cell ratio and the TIM3^+^CD8^+^ T-cell ratio in lung cancer patients. **(E)** Correlation between the PD1^+^CD8^+^T-cell ratio and CD39^+^CD8^+^ T-cell ratio in lung cancer patients. **(F)** Correlation between the PD1^+^CD8^+^ T-cell ratio and IL-6 expression in lung cancer patients. **(G)** Heatmap analysis of the correlation between IL-6 and PD1, TIM3, NRP1, CD352, and CD39 in patients with lung cancer. ns, not statistically significant. *P < 0.05, **P < 0.01, ***P < 0.001.

To further validate the status of CD8^+^ T cells in the TME, we analyzed NSCLC single-cell sequencing data (EMTAB6149) from the TISCH database. The data of 40,218 cells from 5 patients were included in this analysis. We found that different immune cells were distributed in different locations, and the proportion of exhausted CD8^+^ T cells (CD8 Tex) and CD8^+^ T cells in the total immune cells was 19.12% and 6.20%, respectively (**Figure 4A**). This finding indicated that the majority of CD8^+^ T cells in tumor patients were exhausted. We also found high expression of CD352, TIM3, CD39, and PD1 on CD8 Tex; this finding is consistent with our previous findings and confirms that our selection of these immune molecules is associated with the exhaustion of CD8^+^ T cells (**Figure 4B**).

Immunotherapies targeting the PD1/PDL1 axis, which uses the body’s own immune system to resist and fight cancer, have been highly successful over the past decade. However, only a small portion of patients showed favorable clinical responses. The majority of patients cannot benefit from nivolumab/PDL1 therapy. IL-6, as a classic proinflammatory factor, may be associated with resistance to immunotherapy. We compared PD1 expression and the correlation between CD352, TIM3, and CD39 in peripheral blood PBMCs and CD8^+^ T cells collected from tumor patients. The results showed that the frequency of PD1^+^CD8^+^ T cells was negatively correlated with CD352^+^CD8^+^ T cells (**Figure 4C**) and positively correlated with TIM3^+^CD8^+^ T cells and CD39^+^CD8^+^ T cells (**Figures 4D, E**). Furthermore, an exploratory comparison revealed that IL-6 was positively correlated with PD1, TIM3, NRP1, and CD39 and negatively correlated with CD352 in the TME, with significant differences (**Figures 4F, G**).

### 3.5 IL-6 positively correlates with tumor-associated pathways

**Figure 5:**
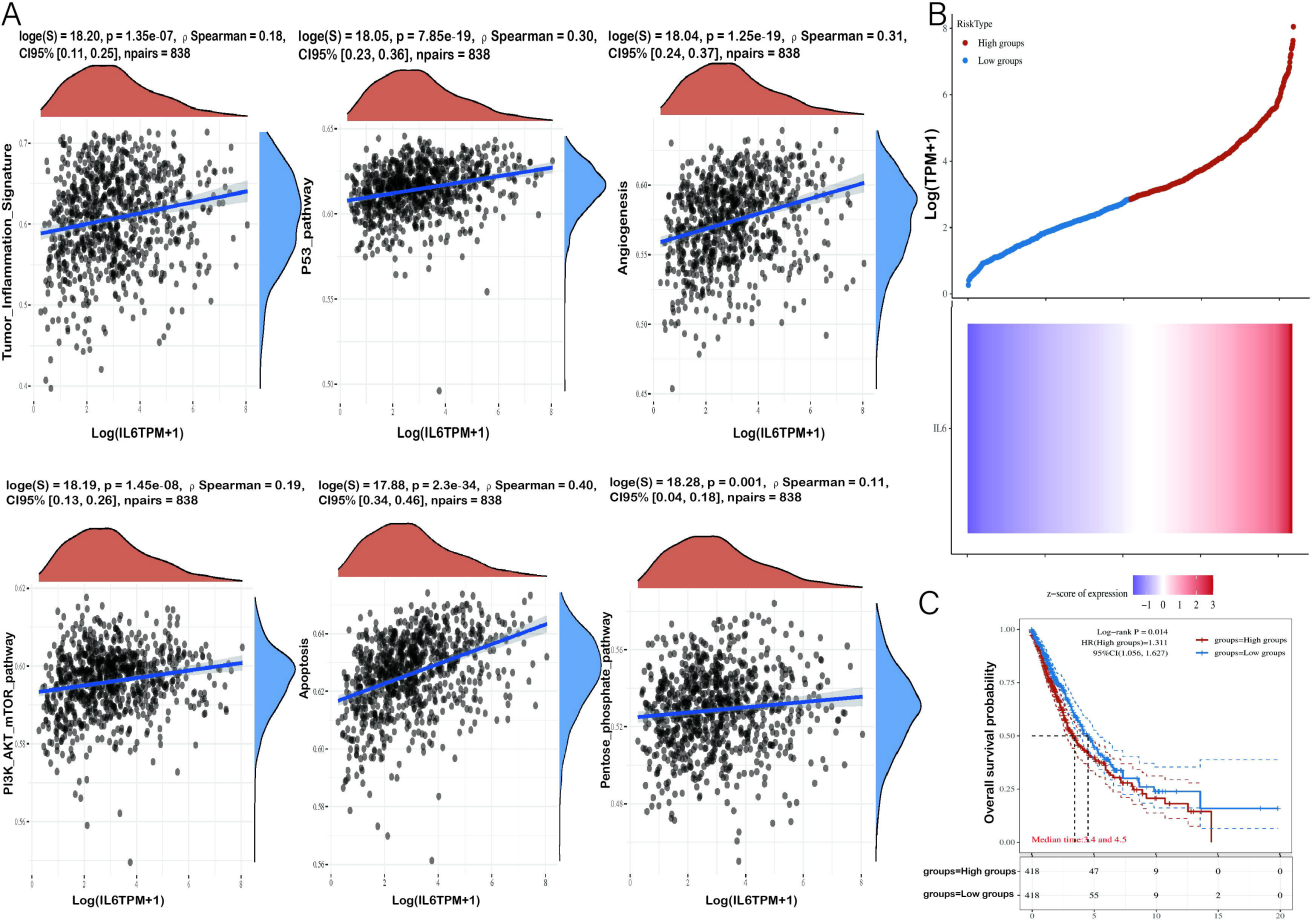
Correlation analysis of IL-6 gene with tumor-related pathways and prognosis. **(A)** The X-axis represents the distribution of IL-6 gene expression and the Y-axis represents the distribution of tumor signaling pathway scores. The density curves on the right side represent the distribution trend of pathway scores, and the density curves on the top side represent the distribution trend of IL-6 expression. The top values represent the results of Spearman’s correlation analysis, including p-value, correlation coefficient, and correlation calculation method. **(B)** The Relationship between gene expression and survival time and survival status of tumor patients in TCGA data. the upper graph represents the scatter plot of IL-6 expression from low to high, with different colors representing different expression groups; the lower graph represents the heat map of gene expression. **(C)** KM survival curves of the gene in TCGA data, where different groups were examined using log-rank. HR (High exp) refers to the risk coefficient of the samples in the high-expression group relative to the samples in the low-expression group. If HR > 1, the gene is a risk factor (the higher the expression, the worse the prognosis). If HR < 1, the gene is a protective factor (higher expression, better prognosis). 95% CI indicates the confidence interval of HR; median time indicates the time corresponding to the survival rate of the high expression group and the low expression group at 50% of the time. e., Median survival time in years.

To investigate the relationship between IL-6 and tumor cell proliferation in NSCLC, we found that IL-6 was positively correlated with a series of tumor-associated pathways by TCGA. Analysis of the database revealed that IL-6 was positively correlated with angiogenesis, tumor inflammatory signaling, apoptosis, Pentose/phosphate/pathway, PI3K/AKT/mTOR pathway, and P-53 pathway (**Figure 5A**). Low expression of IL-6 was significantly associated with a longer overall survival in NSCLC (p < 0.05, HR > 1), the results suggest that IL-6 is a risk factor, and the higher its expression, the worse the prognosis. (**Figures 5B, C**). Moreover, COPS6 was found to be not only a mediator of IL-6 secretion in the tumor microenvironment, but also a negative regulator of CD8^+^T cells tumor infiltration in breast cancer. By regulating IL-6 secretion, p53/COPS6 reduces the infiltration and function of CD8^+^ T cells, thus promoting the progression of breast cancer (27). Based on single-cell RNA sequencing, circulating CTLs from cancer patients with high plasma levels of IL-6 show suppressed functional characteristics, and IL-6-STAT3 signaling inhibits classical cytotoxic differentiation of CTLs *in vitro*. In homozygous mice, CTL-specific IL6R deficiency further enhances anti-PD-L1 therapy. Therefore, based on clinical and experimental evidence, drugs targeting IL-6 signaling are reasonable partners for cancer patients in combination therapy with immune checkpoint inhibitors (28).

### 3.6 IL-6 stimulation *in vitro* promotes enhanced CD8^+^ T cell exhaustion

**Figure 6:**
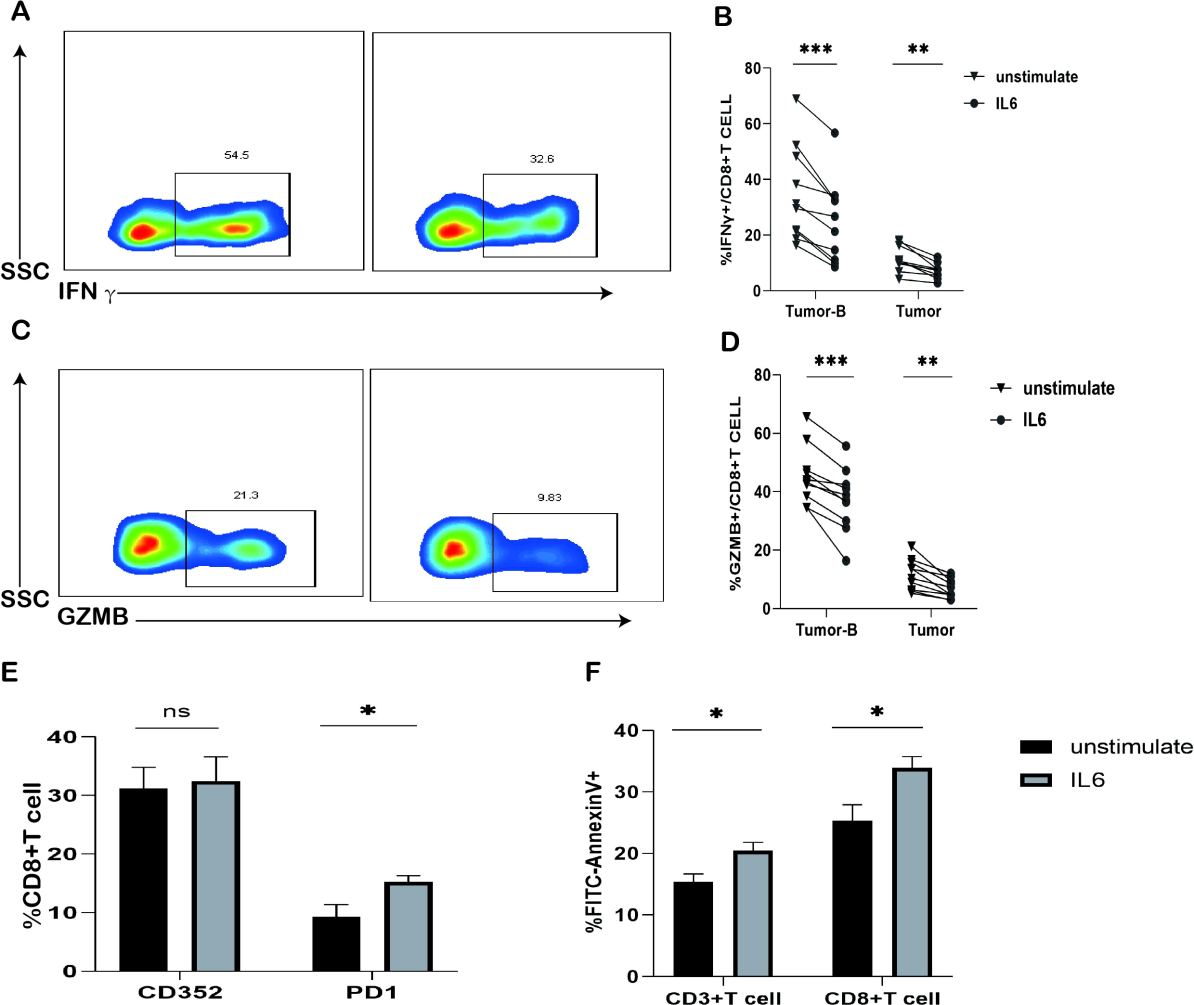
Effects of IL-6 *in vitro* stimulation on T cells. **(A)** Typical graph of reduced GZMB expression in CD8^+^ T cells in the Tumor-B and Tumor groups after 24 h of IL-6 treatment. **(B)** Frequency graph of reduced IFN-γ expression in CD8^+^ T cells of the Tumor-B and Tumor groups after 24 h of IL-6 treatment. **(C)** Typical graph of reduced GZMB expression in CD8^+^ T cells of the Tumor-B and Tumor groups after 24 h of IL-6 treatment. **(D)** Frequency graph of reduced GZMB expression in CD8^+^ T cells of the Tumor-B and Tumor groups after 24 h of IL-6 treatment. **(E)** Frequency graph of CD8^+^ T cells in the peripheral blood after stimulation with the IL-6 recombinant protein **(F)** Increased apoptosis of CD3^+^ T cells and CD8^+^ T cells in the peripheral blood after IL-6 treatment for 24 h. Note: Tumor-B and Tumor groups were the peripheral blood PBMC group and the cancer tissue group of patients with non-small cell lung cancer, respectively. ns, not statistically significant. *P < 0.05, **P < 0.01, ***P < 0.001.

To explore whether alterations in the TME can influence the functionality of CD8^+^ T cells, we examined the impact of IL-6 on CD8^+^ T cell function. We isolated peripheral blood PBMCs (Tumor B) and obtained single-cell suspensions from tumor tissues (Tumor) of lung cancer patients. Subsequent to stimulation with recombinant IL-6 protein for 24 h, we detected the secretion of GZMB and IFN-γ by CD8^+^ T cells when stimulated with PMA/ionomycin containing Mullenicin. It was observed that both the Tumor B and Tumor groups exhibited reduced levels of GZMB and IFN-γ (**Figures 6A-D**), indicating that IL-6 can diminish the effector killing capacity of CD8^+^ T cells.

Furthermore, we investigated whether IL-6 stimulation affected the expression of CD352 and PD1 on CD8^+^ T cells. After inducing T cell activation *in vitro* with anti-CD3/anti-CD28 antibodies, we found that IL-6 stimulation notably increased the frequency of PD1 expression on CD8^+^ T cells (**Figure 6E**), although it did not significantly impact the frequency of CD352 expression on CD8^+^ T cells. Additionally, there was a significant rise in the proportion of apoptosis among CD3^+^ T cells and CD8^+^ T cells in the presence of high concentrations of IL-6 (**Figure 6F**). These findings suggest that IL-6 may be among the factors responsible for inducing exhaustion in CD8^+^ T cells.

### 3.7 Restoration of CD8^+^ T cell function after the combined blockade of PD1 and IL-6

**Figure 7:**
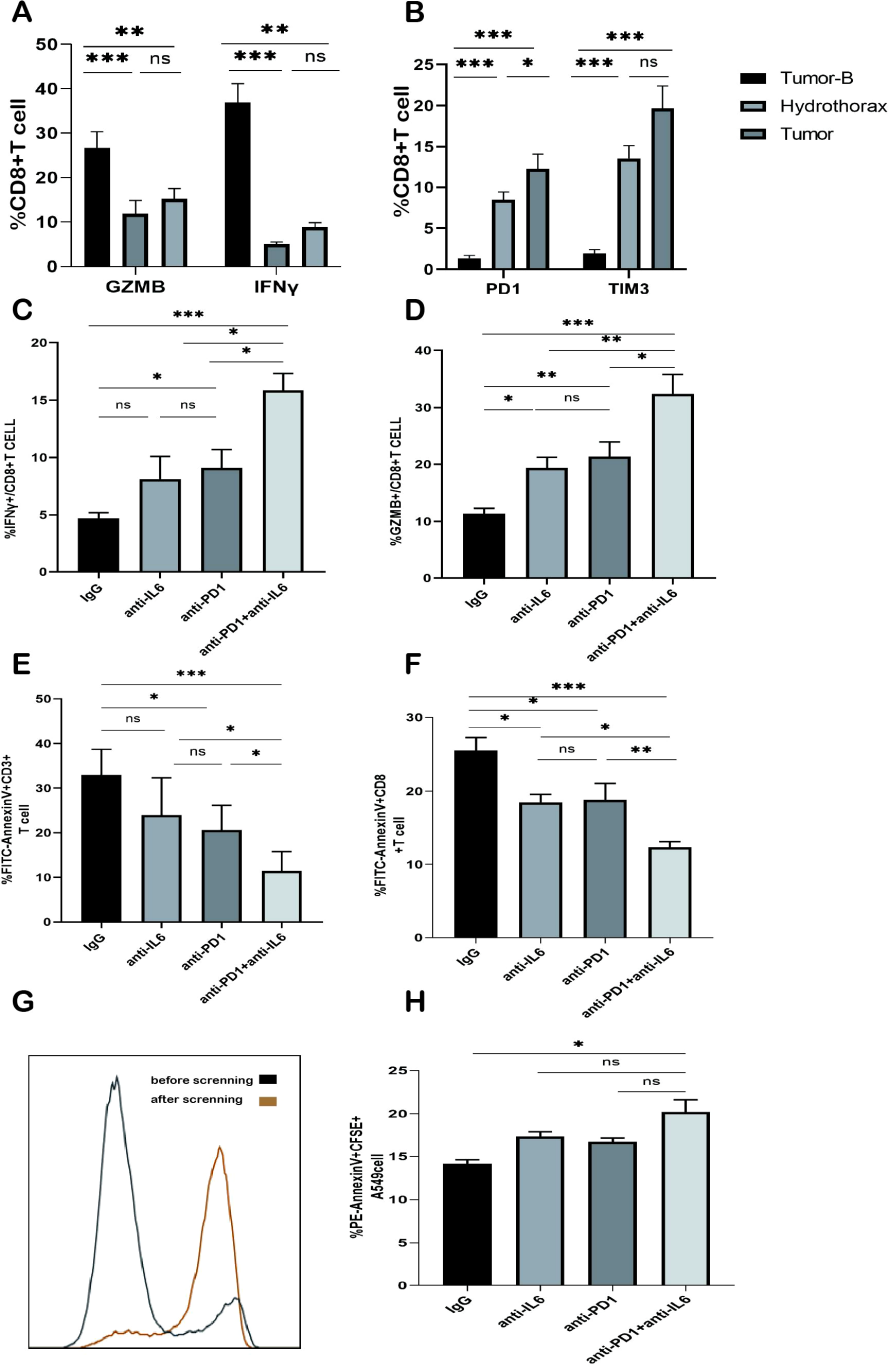
Effect of the combined blockade of PD1 and IL-6 on CD8^+^ T cells *in vitro*. **(A)** Frequency plot of IFN-γ and GZMB expression in CD8^+^ T cells of the Tumor-B, Hydrothorax, and Tumor groups. **(B)** Frequency plot of CD8^+^ T cells expressing PD1 and TIM3 in the pleural effusion of the Tumor-B group. **(C)** Frequency of IFN-γ expression in the pleural effusion of lung cancer patients after anti-PD1 and/or anti-IL-6 blockade. **(D)** Frequency of GZMB expression in the pleural effusion of lung cancer patients after anti-PD1 and/or anti-IL6 blockade. **(E)** Frequency of CD3^+^ T cell apoptosis in the pleural effusion of lung cancer patients after PD1 and/or IL-6 blockade. **(F)** Frequency of CD8^+^ T cell apoptosis in the pleural effusion of lung cancer patients after PD1 and/or IL-6 blockade. **(G)** CD8^+^ T cells before and after magnetic bead sorting. **(H)** Typical map and expression frequency map of A549 cell apoptosis in the pleural effusion of lung cancer patients after the blockade with PD1 and/or IL-6; ns, not statistically significant. *P < 0.05, **P < 0.01, ***P < 0.001.

Finally, we investigated whether the combined nivolumab and anti-IL-6 blockade could enhance the function of CD8^+^ T cells. We collected fresh pleural effusion samples from patients with NSCLC and isolated lymphocytes from these samples. This is because CD8^+^ T cells in the pleural effusion showed a significantly reduced ability to produce IFN-γ and GZMB as compared to peripheral blood lymphocytes. Interestingly, PD1 and TIM3 expression on the surface of CD8^+^ T cells in pleural effusion was further increased as compared to that on CD8^+^ T cells in peripheral blood; however, the difference was not significant when compared with the expression on CD8^+^ T cells in tumor tissue suspension. This finding suggested that CD8^+^ T cells in pleural effusions of tumor patients and CD8^+^ T cells in the tumor tissue were in a severe state of functional exhaustion (**Figures 7A, B**). We evaluated the capacity of CD8^+^ T cells from the pleural effusion sample to secrete IFN-γ and GZMB following 3 days of CD3/CD28 stimulation. While PD1 blockade alone reinstated the ability of CD8^+^ T cells to produce IFN-γ and GZMB compared to the isotype control IgG, the combination of PD1 and IL-6 monoclonal antibodies yielded significantly higher levels of IFN-γ and GZMB expression (**Figures 7C, D**). Furthermore, it was observed that the combination of nivolumab and anti-IL-6 antibodies effectively inhibited the apoptosis of both CD3^+^ T cells and CD8^+^ T cells (**Figures 7E, F**). These findings further underscore the prognostic significance of CD8^+^ T cells in NSCLC and the association between achieving potent antitumor effects by targeting the PD1 inhibition pathway, especially when combining PD1 blockade with anti-IL-6 antibodies.

Our previous results showed that the combined anti-IL-6 and nivolumab blockade reduced CD8^+^T cell exhaustion. Next, we investigated the effect of the combined treatment on CD8^+^ T cell function. We first isolated peripheral blood PBMCs from healthy volunteers and purified CD8^+^ T cells by magnetic bead sorting (**Figure 7G**). The purified CD8^+^ T cells were activated by stimulation with CD3/CD28 antibodies and then co-cultured with CFSE-labeled A549 cells in the presence of anti-IL-6 and/or nivolumab antibodies or IgG for 48 h. A549 cell apoptosis was analyzed by flow cytometry. The apoptosis rate of A549 cells in the combined IL-6 and PD1 blocked group was significantly increased (**Figure 7H**); thus, indicating that the tumor cell killing function of CD8^+^T cells was enhanced. These results illustrated that the combined treatment of IL-6 and PD1 blockade could enhance the antitumor immune function of CD8^+^ T cells in NSCLC and restore their effector killing function.

### 3.8 Combination of nivolumab and anti-IL-6 antibodies recruits macrophages

**Figure 8:**
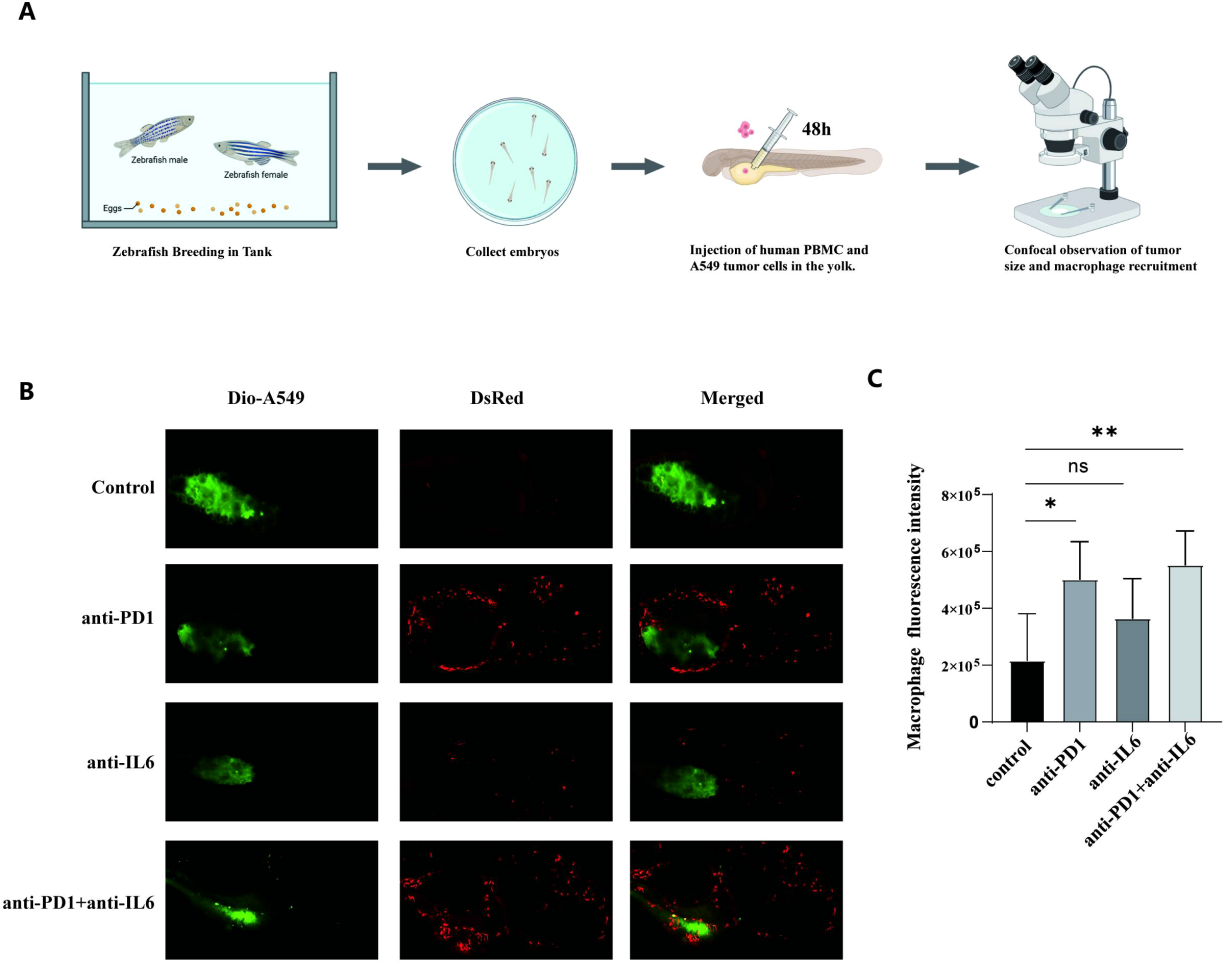
Macrophage recruitment by combination treatment with nivolumab and anti-IL-6 antibody. **(A)** The process of zebrafish model construction. **(B)** Comparison of zebrafish macrophage fluorescence between control and experimental groups. **(C)** Statistical analysis of zebrafish macrophage fluorescence intensity between control and experimental groups (mean ± SD, n = 5). ns, not statistically significant. *P < 0.05, **P < 0.01.

In zebrafish, we evaluated the effects of anti-PD1, anti-IL-6, and a combination of anti-PD1 and anti-IL-6 on macrophage recruitment using the cell line A549 (**Figure 8A**). Zebrafish inoculated with Dio-A549 cells and PBMC were injected intravenously with different antibody groups and changes in fluorescence were recorded. The anti-PD1 and anti-IL6 combination treatment group significantly increased the number of macrophages compared to the control group. (**Figures 8B, C**). The results suggested that the combination treatment recruited macrophages better *in vivo*.

### 3.9 Combination of nivolumab and anti-IL-6 antibodies inhibits tumor growth in PBMC humanized mice

**Figure 9:**
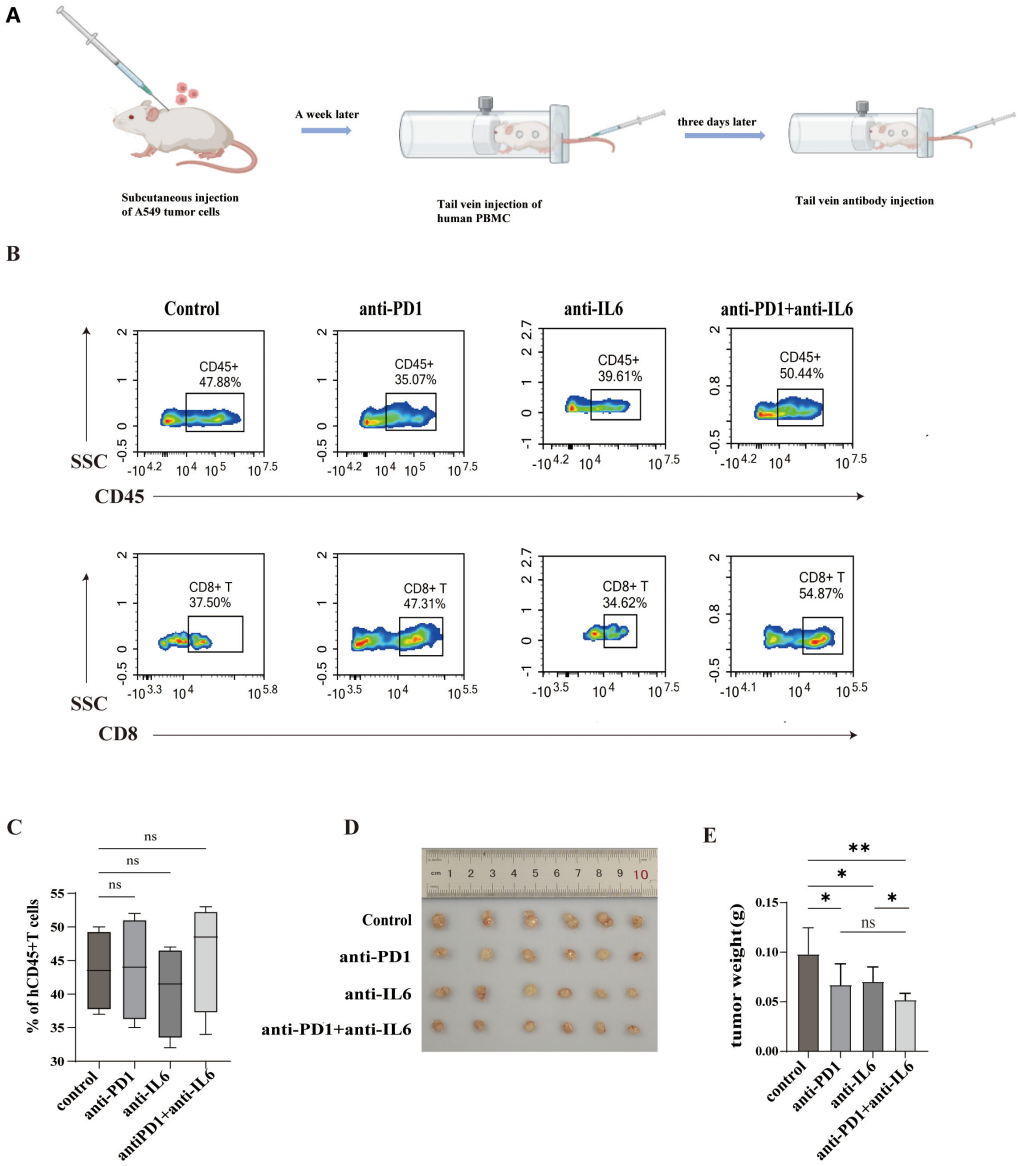
Combined blockade of PD1 and IL-6 on humanized mouse tumors. **(A)**Construction of a humanized hormonal mouse model and process of antibody administration. **(B, C)** Flow cytometry assays were performed to assess mouse peripheral blood HuCD45^+^T cells and HuCD8^+^T cells and their proportions. **(D)** Comparison of tumors in control and experimental mice. **(E)** Tumor weights of control and experimental mice. ns, not statistically significant. *P < 0.05, **P < 0.01.

The construction of PBMC humanized mouse tumor model and the process of antibody administration are shown in **Figure 9A**. The changes in the proportion of HuCD45^+^T and HuCD8^+^T cells in the peripheral blood of mice were detected by flow cytometry, and the proportion of HuCD45^+^T in the peripheral blood of the mice > 25% signified the successful construction of the humanized model. The proportion of HuCD45^+^T in the peripheral blood of both control and experimental mice was > 25% without significant difference, indicating a similar degree of immune reconstitution in the mice (**Figures 9B, C**).

Our experiments revealed that in humanized mouse tumor models, nivolumab and anti-IL-6 antibody alone as well as the combination of nivolumab and anti-IL-6 antibody inhibited the growth of mouse tumors, but the combination treatment was more effective in controlling tumor growth (**Figures 8D, E**) and improved mouse survival. This suggests that the combination therapy has a better anti-tumor effect *in vivo*.

## 4 Discussion

The therapeutic potential of combination immunotherapy is currently being evaluated in early clinical trials. However, there are many unknown factors regarding the biological and clinical significance of these receptors; the evaluation of these factors is crucial for the rational design of immunotherapy (29, 30). Cytotoxic CD8^+^ T cells infiltrating tumors specifically inhibit tumor growth, but tend to become “exhausted” or “dysfunctional” (31).

To analyze how CD8^+^ T cell exhaustion causes failure of immune regulation during NSCLC pathogenesis, we investigated the status and function of CD8^+^ T cells from PBMCs in the peripheral blood of healthy individuals and PBMCs in the peripheral blood of tumor patients, tumor tissues, and peri-tumor tissues. We also detected cytokines in plasma and tumor tissue supernatants; this could help investigate immunosuppressive mechanisms in the TME of NSCLC. We found that although the proportion of infiltrating CD8^+^ T cells was significantly increased in tumor tissues, they highly expressed immunosuppressive molecules, such as PD1, accompanied by the dysfunction of secretion of cytotoxic factors. This finding indicated that the infiltrated CD8^+^ T cells in the tumor tissue were in the state of exhaustion; moreover, *in vitro* stimulation of IL-6 elevated the dysfunction of CD8^+^ T cells and facilitated increased exhaustion of CD8^+^ T cells. Furthermore, the levels of the inflammatory cytokines IL-6, IL-7, and IL-17A were differentially elevated in plasma and tissue supernatants. The combined blockade therapy using anti-IL-6 and nivolumab antibodies restored the function of CD8^+^ T cells and induced the exhaustion of CD8^+^ T cells in the TME.

The levels of CD3^+^ T cells and CD8^+^ T cells were significantly elevated in the tumor tissues of NSCLC patients and further analyzed the reasons for the elevation of effector T cells without tumor suppression. We also observed abnormal elevation of TIM3, CD39, NRP1, and PD1 expression on infiltrating CD8^+^ T cells in tumor tissues. The function of CD8^+^ T cells in tumor tissues was also altered, with a significantly lower level of secretion of IFN-γ and granzyme B. This resulted in the over-exhaustion of CD8^+^ T cells in the TME. The analysis of the TISCH single-cell data showed that the majority of CD8^+^T cells in NSCLC were in the exhausted state; the results also confirmed the elevated expression of TIM3 and PD1 on the surface of exhausted T cells.

T cells are not exhausted at the beginning; however, they gradually move toward exhaustion under long-term stimulation by tumor antigens, which is a dynamic process (32, 33). Previous studies have categorized exhausted T cells into initial and terminal exhausted subpopulations. The exhausted CD8^+^T cell subsets were categorized mostly based on PD1 expression levels, with a moderate level of expression of PD1 on CD8^+^T cells as the initial exhausted subset and a high level of expression of PD1 as the terminal exhausted subset (34). However, in the present study, the CD352 expression level was significantly higher in PBMCs from tumor patients than in peripheral blood PBMCs from healthy volunteers, but decreased with proximity to the TME tissues; this was accompanied by high expression of immunosuppressive molecules such as CD39, TIM3, and PD1 and a decrease in effector killer cytokines. CD352, also known as SLAMF6, is a homotypic binding receptor expressed on both resting and activated PBMC, and its absence significantly ameliorated CD8^+^T cells mediated tumor regression, suggesting that it serves as an inhibitory checkpoint. When it was absence, CD8^+^T cells were more responsive to tumor cells. The strong effector trait is attributed to a series of T-bet-mediated transcriptional events, and in the absence of SLAMF6, T-bet-regulated pathways may play a role in the generation of “type 1” effector cells with inflammatory features and high cytotoxicity in CD8^+^ T cells (35, 36). Blockade of SLAMF6 decreased the number and proportion of PD1^+^CD3^+^CD44^+^CD8^+^T cells and enhanced cytotoxicity. Possible reasons for reduction in exhausted PD1^+^CD8^+^ T cells include induction of ADCC and/or downregulation of PD-1 from the cell surface after SLAMF6 ligation (37).The above studies mainly focused on mice, in this study we found that the level of CD352 was significantly higher in PBMC from tumor patients compared to peripheral blood PBMC from healthy volunteers, but with the proximity of the tumor microenvironment, the expression level of CD352 was reduced, which was accompanied by a high level of expression of immunosuppressive molecules, such as CD39, TIM3, and PD1, as well as a decrease in effector killer cytokines. Therefore, we believe that CD352 expressed on CD8^+^T cells in NSCLC can be used as a marker of initial exhaustion, and most of the CD8^+^T cells in the peripheral blood of tumor patients are in the state of initial exhaustion, whereas PD-1^+^TIM3^+^CD8^+^T cells within the tumor microenvironment are in the state of terminal exhaustion with increased immunosuppressive molecules and severe dysfunction of killing function.

Another noteworthy discovery is that elevated IL-6 levels might constitute a significant factor contributing to CD8^+^ T cell exhaustion. IL-6 is a versatile cytokine with an extensive array of biological functions, and it is released by various cell types, including fibroblasts, macrophages, T and B lymphocytes, endothelial cells, and activated keratinocytes (38, 39). Stimulation with IL-6 can prompt cell proliferation, enhance cell survival, and increase the invasiveness of tumor cells. Moreover, high levels of IL-6 are associated with the severity of the disease and an unfavorable clinical prognosis (40, 41).

Our results showed that IL-6, IL-7, IL-10, and IL-17A levels were elevated in the plasma supernatants of NSCLC patients as compared to those in healthy controls but were significantly lower than those in the tumor tissue supernatant; this finding suggests an increased inflammatory response in the TME. A significant increase was noted in IL-6 expression levels in the TME; however, the relationship between IL-6 expression levels and T cell exhaustion in the TME has not been well studied. According to previous studies, IL-6 in combination with IL-27 can promote CD39 expression on CD8^+^T cells; however, the specific role of IL-6 in CD8^+^ T cell exhaustion has not been analyzed.

We first integrated the data by correlation regression analysis and found that IL-6 expression was positively correlated with PD1, TIM3, CD39, and NRP1 and negatively correlated with CD352. Hence, we suggest that IL-6 may be associated with the promotion of T cell exhaustion. *In vitro* stimulation experiments showed that PD1 expression on the surface of CD8^+^ T cells was increased and the ability to secrete IFN-γ and GZMB was decreased by stimulation with high concentrations of IL-6; however, IL-6 stimulation had no significant effect on CD352 expression on the surface of CD8^+^ T cells. This finding suggests that IL-6 further deepened the exhaustion of CD8^+^ T cells and the exhaustion subset of T cells mainly depends on the terminal exhausted T cells with a weaker effector killing function. IL-6 also promotes increased apoptosis of CD3^+^ T cells and CD8^+^ T cells; thus, further exacerbating immunosuppression.

Our previous studies had found that IL-8 was also highly expressed in the tumor microenvironment, and high levels of IL-8 were significantly associated with poor prognosis in advanced NSCLC patients treated with HFRT (hypofractionated radiotherapy) PD1 blockade, and that high circulating IL-8 in NSCLC increased apoptosis of effector memory T cells and CD8^+^T cells (42). By applying an in-silico multidimensional model integrating spatially resolved and single-cell gene expression data to show that release of IL-10 promotes T cell exhaustion, thereby contributing to the immunosuppressive tumor microenvironment (43). The extended half-life IL10-Fc fusion protein effectively enhanced expansion and effector function of terminally exhausted CD8^+^ T cells by promoting oxidative phosphorylation (44). IL-17 promotes terminal exhaustion of CD8^+^T cells and tumor progression *in vivo*, which can be reversed by blocking either the IL-17 or RORγt pathway. These findings reveal novel roles for the production of IL-17 as a promoter of CD8^+^T cells exhaustion and suggest IL-17 as a promising target for cancer immunotherapy (45), The above studies in combination with our experiments have demonstrated that cytokines such as IL-10 or IL-17 have the potential to restore the function of exhausted CD8^+^T cells. Combined blockade of IL-6 or IL-10 or IL-17 may better improve the prognosis of tumor patients.

Currently, only a minority of patients with advanced NSCLC benefit from PD1/PD-L1 antibody therapy. It has been found that PD1/PD-L1 combined with hypofractionated radiotherapy (HFRT) or IL-8 inhibitors could enhance anti-tumor immune responses (42, 46). The combination of ICIs (e.g., PD1 blockers) with cytokine antibodies (e.g., IL-6) could be a reasonable approach to improve the efficacy of immunotherapy for NSCLC. We observed significantly higher expression of PD1 in tumor-infiltrating CD8^+^ T cells. Blocking with IL-6 or PD1 alone enhanced CD8^+^ T cell toxicity in NSCLC patients and reduced cell apoptosis. Following the combined IL-6 and PD1 blockade, CD8^+^ T cells from NSCLC patients secreted more cytokines and had a lower apoptosis rate than those subjected to single agent blocking. This finding suggests that the combined blockade of IL-6 and PD1 promotes functional recovery of exhausted CD8^+^ T cells. We screened purified CD8^+^ T cells from healthy volunteers and co-cultured CD3/CD28-activated CD8^+^T cells with tumor cells. The results showed a significant increase in tumor cell apoptosis after combined IL-6 and PD1 blockade. In a PBMC humanized mouse tumor model, mouse tumors were also better suppressed after combination blockade. Similar to all immunotherapies, it is critical to assess the potential toxicity of combination therapies. A limitation of the present study is that animal studies were not conducted to demonstrate toxicity of IL-6- and PD1-targeted therapies. It should be noted that the Adi Diab team conducted a Phase II clinical trial (NCT04940299) to evaluate the safety and efficacy of tocilizumab in combination with ipilimumab and nivolumab for treating primary metastatic melanoma, NSCLC, and urothelial carcinoma (47). One of the limitations of this study is that there was no further combined blockade of IL-10 or IL-17 to assess the efficacy of combined blockades in the treatment of malignant tumors. Follow-up studies in this area will continue.

In summary, CD8^+^ T cells are the key cells in the antitumor immune response and are currently the primary target of immunotherapy. Enhancing the function of exhausted CD8^+^ T cells is associated with improved prognosis and beneficial responses to immune checkpoint blockade. Here, we evaluated the phenotype and function of CD8^+^ T cells in NSCLC patients and proposed that CD8^+^ T cells are in an exhaustion state in patients with NSCLC. High IL-6 levels are one of the main factors that promote T cell exhaustion. In our preliminary study, the combined blockade of IL-6 and PD1 enhanced the killing function of exhausted CD8^+^ T cells; this finding provides a good direction for future research on exhausted T cells. In summary, our results revealed the important roles of IL-6 and PD1 in influencing T cell exhaustion; furthermore, the combined blockade of IL-6 and PD1 could be a potential therapeutic approach for the immunotherapy of patients with NSCLC.

## Data Availability

The original contributions presented in the study are included in the article/**Supplementary Material**. Further inquiries can be directed to the corresponding authors.
