# Supplementary material for: Analysis of tumor-infiltrating exhausted T cells highlights IL-6 and PD1 blockade as a combined immunotherapy strategy for non-small cell lung cancer
Source: Front Immunol. 2025 Feb 11;16:1486329. doi: 10.3389/fimmu.2025.1486329 (PMC11876966; doi:10.3389/fimmu.2025.1486329)
Supplement: Supplementary file 1 [file DataSheet1.docx]

Supplementary Information

We analyzed the correlation between IL-6 and CD352 in lung cancer using the GEPIA2 database and found that IL-6 was significantly correlated with CD352. Meanwhile, there was also an exhausted CD8^+^T cell subpopulation in the GEPIA2 database, and this subpopulation included a total of six exhaustion-related molecules, including HAVCR2, TIGIT, LAG3, PDCD1, CXCL13, and LAYN, and IL-6 was also significantly correlated with this subpopulation. Therefore, we suggest that the negative effect of IL-6 may accompany the whole process of CD8^+^T exhaustion （supplementary material）. Later, we will further explore the role and changes of IL-6 in the process of CD8^+^ T cell exhaustion.


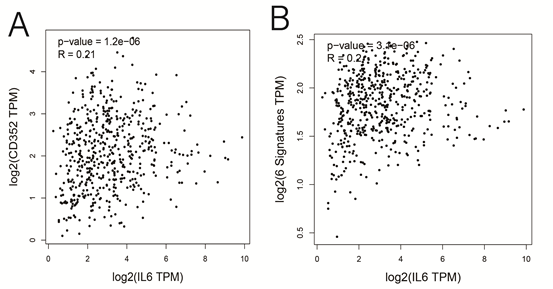


Supplementary Figure 1

Figure (A) shows the correlation between IL-6 and CD352 in tumor tissues and pericarcinoma tissues of patients with non-small cell lung cancer; and Figure (B) shows the correlation between IL-6 and exhausted CD8+ T cell subsets in tumor tissues and pericarcinoma tissues of patients with non-small cell lung cancer.

Table 1.

| **Flow cytometry antibodies** | **Firm** |
| --- | --- |
| FITC anti-human CD3 Antibody | BioLegend (USA) |
| PE/Cyanine7 anti-human CD3 Antibody | BioLegend (USA) |
| APC-Cy™7 anti-Human CD8 Antibody | BioLegend (USA) |
| PerCP/Cyanine5.5 anti-human CD8 Antibody | BioLegend (USA) |
| APC anti-human PD1 Antibody | BioLegend (USA) |
| PE Anti-Human CD352 Antibody | BioLegend (USA) |
| PE Anti-Human TIM3 Antibody | BioLegend (USA) |
| 7-AAD Viability Staining Solution | BioLegend (USA) |
| PE/Cyanine7 anti-human NRP1 Antibody | BioLegend (USA) |
| Human TruStain FcX™(Fc B Receptor locking Solution) | BioLegend (USA) |
| Cell activation cocktial(with brefeldin A) | BioLegend (USA) |
| APC Anti-Human CD39 Antibody | BioLegend (USA) |
| Hu IFN-Gma PE-Cy7 | BD Bioscience (USA) |
| Hu Granzyme B PE | BD Bioscience (USA) |
| Human IL-6 Flex Set | BD Bioscience (USA) |
| Human IL-10 Flex Set | BD Bioscience (USA) |
| Human IL-17A Flex Set | BD Bioscience (USA) |
| Human TNF Flex Set | BD Bioscience (USA) |
| Alexa Fluor® 647 Conjugate | Cell Signaling Technology(USA) |
| Alexa Fluor® 594 Conjugate | Cell Signaling Technology(USA) |
| TCF1/TCF7(C63D9)Rabbit mAb | Cell Signaling Technology(USA) |
| Tox/Tox2(E6G5O)Rabbit mAb | Cell Signaling Technology(USA) |

Table 2.

| Primer name | Base sequence |
| --- | --- |
| GAPDH | Forward: 5'-CATCCTGGGCTACACTGAGC-3'  Reverse : 5'-AAAGTGGTCGTTGAGGGCAA-3' |
| EMOES | Forward: 5'-GTGGCAAAGCCGACAATAAC-3'  Reverse : 5'-CCGAATGAAATCTCCTGTCTCA-3' |
| IL-6 | Forward:5'-ATGAACTCCTTCTCCACAAGCGC-3'  Reverse: 5'-GGGAAGGCAGCAGGCAACAC-3' |
| T-BET | Forward: 5'-AAGGATTCCGGGAGAACTTTG-3'  Reverse:5'-GTTGGGTAGGAGAGGAGAGTAG-3' |
| TCF1 | Forward:5'-GTGATGAGCTACCAACCAAGAA-3'  Reverse: 5'-AGGGTTCTTCTGCCTCTCATA-3' |

Table 3.

| **Antibody** | **Manufacturer** | **Cat no.** | **Dilution used** | **species** | **Observed band size** |
| --- | --- | --- | --- | --- | --- |
| TCF1/TCF7(C63D9)Rabbit mAb | Cell Signaling Technology | 2203T | 1:1000 | Rabbit mAb | 48,50kDa |
| Tox/Tox2(E6G5O)Rabbit mAb | Cell Signaling Technology | 36778s | 1:1000 | Rabbit mAb | 60-80kDa |
